# Supplementary figures and images for: Close neighbors, not intruders: investigating the role of tank bromeliads in shaping faunal microbiomes
Source: PeerJ. 2025 May 9;13:e19376. doi: 10.7717/peerj.19376 (PMC12068248; doi:10.7717/peerj.19376)

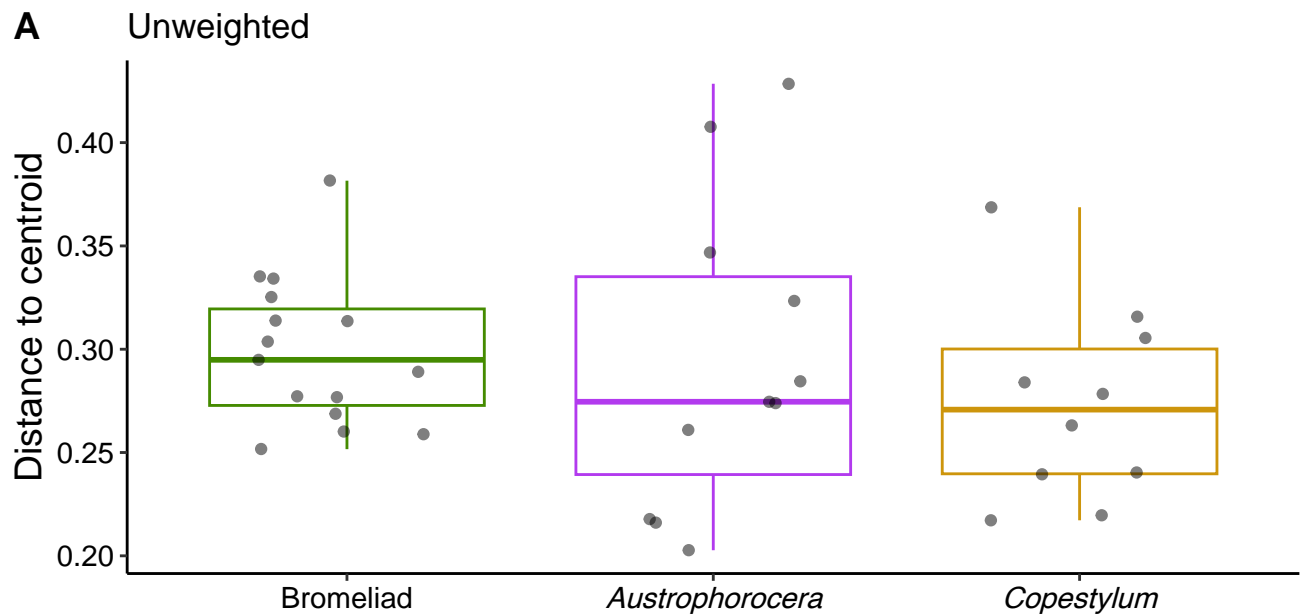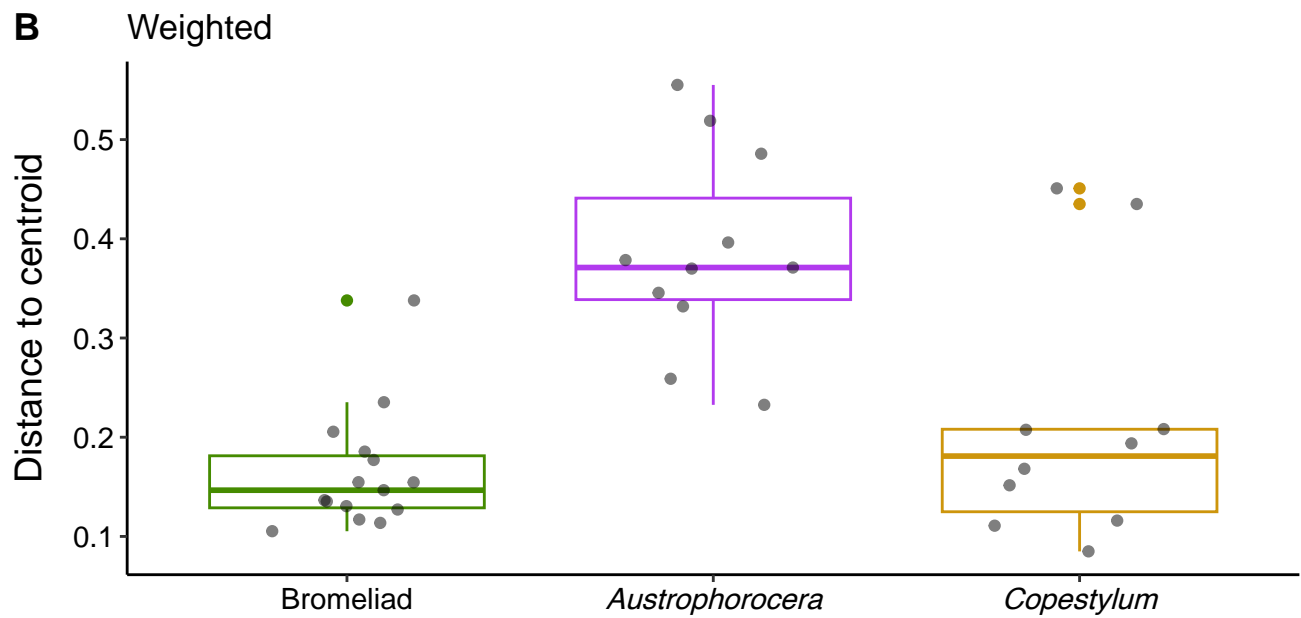

Supplement: Supplemental Information 1 — (A) Data based on unweighted UniFrac distances. (B) Data based on weighted UniFrac distances. [file peerj-13-19376-s001.pdf]
