# Supplementary material for: Close neighbors, not intruders: investigating the role of tank bromeliads in shaping faunal microbiomes
Source: PeerJ. 2025 May 9;13:e19376. doi: 10.7717/peerj.19376 (PMC12068248; doi:10.7717/peerj.19376)
Supplement: Supplemental Information 4 [file peerj-13-19376-s004.zip › bromeliads-scripts-and-working-files/qiime2_code/1.code-to-run-qiime2.rtf]

QIIME2 CODE FOR PROCESSING 16S SEQUENCES FROM TANK BROMELIADS AND FLIES## QIIME2 VERSION: qiime2-amplicon-2023.9##Importing samples with Casava 18: qiime tools import \--type 'SampleData[PairedEndSequencesWithQuality] ' \--input-path Bromeliads_flies \--input-format CasavaOneEightSingleLanePerSampleDirFmt \--output-path bromeliads-demux-paired-end.qza ##Check quality plotsqiime demux summarize \--i-data bromeliads-demux-paired-end.qza \--o-visualization bromeliads-demux.qzv##Conduct primer trimming with cut adaptqiime cutadapt trim-paired --i-demultiplexed-sequences bromeliads-demux-paired-end.qza --p-front-f ACGCGHNRAACCTTACC --p-front-r ACGGGCRGTGWGTRCAA --p-error-rate 0 --o-trimmed-sequences trimmed-bromeliads-demux-paired-end.qza --verbose##Denoising, merging, chimera removal, and ASV assignation using DADA2#Since primers were already removed, then, --p-trim-left-f and --p-trim-left-r values are 0. Values for --p-trunc-len-f and --p-trunc-len-r were set based on quality plots information. qiime dada2 denoise-paired \--i-demultiplexed-seqs trimmed-bromeliads-demux-paired-end.qza \--p-trim-left-f 0 \--p-trim-left-r 0 \--p-trunc-len-f 225 \--p-trunc-len-r 200 \--o-table table.qza \--o-representative-sequences bromeliads-rep-seqs.qza \--o-denoising-stats bromeliads-denoising-stats.qza##Phylogenetic tree reconstructionqiime phylogeny align-to-tree-mafft-fasttree \  --i-sequences bromeliads-rep-seqs.qza \  --o-alignment aligned-rep-seqs.qza \  --o-masked-alignment masked-aligned-rep-seqs.qza \  --o-tree bromeliads-unrooted-tree.qza \  --o-rooted-tree bromeliads-rooted-tree.qza##Taxonomic classification with SILVA qiime feature-classifier classify-sklearn \  --i-classifier silva-132-99-nb-classifier-full-length-sequences.qza\  --i-reads bromeliads-rep-seqs.qza \  --o-classification bromeliads-taxonomy.qza##Filtering chloroplast and mitochondriaqiime taxa filter-table \  --i-table table.qza \  --i-taxonomy bromeliads-taxonomy.qza \  --p-exclude mitochondria,chloroplast \  --o-filtered-table bromeliads-table-nonchloro.qza##Because the number of sequences differ between bromeliad and flies samples, I split the ASV table (i.e., bromeliads-table-nonchloro.qza) to filter separately low abundant ASVs.#Bromeliads onlyqiime feature-table filter-samples \  --i-table bromeliads-table-nonchloro.qza \  --m-metadata-file bromeliads_flies.txt \  --p-where "Stay IN ('No')" \  --o-filtered-table bromeliads-only-table.qza  #Flies onlyqiime feature-table filter-samples \  --i-table bromeliads-table-nonchloro.qza \  --m-metadata-file bromeliads_flies.txt \  --p-where "Stay IN ('Yes')" \  --o-filtered-table flies-only-table.qza #Bromeliads: Filter ASVs present in 10% of samples and less than 50 reads summed across all samples qiime feature-table filter-features \  --i-table bromeliads-only-table.qza \  --p-min-samples 2 \  --o-filtered-table bromeliads-only-table-morethan2samples.qzaqiime feature-table filter-features \  --i-table bromeliads-only-table-morethan2samples.qza \  --p-min-frequency 50 \  --o-filtered-table bromeliads-only-table-final.qza#Flies: Filter ASVs present in 10% of samples and less than 10 reads summed across all samplesqiime feature-table filter-features \  --i-table flies-only-table.qza \  --p-min-samples 4 \  --o-filtered-table flies-only-table-morethan4samples.qza  qiime feature-table filter-features \  --i-table flies-only-table-morethan4samples.qza \  --p-min-frequency 10 \  --o-filtered-table flies-only-filtered-table-final.qza #Merge the two filtered tables (bromeliads and flies)qiime feature-table merge \  --i-tables bromeliads-only-table-final.qza \  --i-tables flies-only-filtered-table-final.qza \  --o-merged-table merged-table-final.qza##ALPHA DIVERSITY ANALYSES IN QIIME2#Core diversity analysesqiime diversity core-metrics-phylogenetic \  --i-phylogeny bromeliads-rooted-tree.qza \  --i-table merged-table-final.qza \  --p-sampling-depth 2400 \  --m-metadata-file bromeliads_flies.txt \  --output-dir core-metrics-bromeliads-flies-final#Alpha diversity analyses for ASV richness (i.e., observed features) and Faith phylogenetic diversityqiime diversity alpha-group-significance \  --i-alpha-diversity core-metrics-bromeliads-flies-final/observed_features_vector.qza \  --m-metadata-file bromelias_flies.txt \  --o-visualization core-metrics-bromeliads-flies-final/observed-otus-significance.qzv  qiime diversity alpha-group-significance \  --i-alpha-diversity core-metrics-bromeliads-flies-final/faith_pd_vector.qza \  --m-metadata-file bromelias_flies.txt \  --o-visualization core-metrics-bromeliads-flies-final/faith_pd-significance.qzv  #Code to obtain distance matrices from qiime2 artifacts#Unweighted UniFracqiime tools export \  --input-path core-metrics-bromeliads-flies-final/unweighted_unifrac_distance_matrix.qza\  --output-path core-metrics-bromeliads-flies-final/unweighted_unifrac_distance_matrix#Weighted UniFracqiime tools export \  --input-path core-metrics-bromeliads-flies-final/weighted_unifrac_distance_matrix.qza\  --output-path core-metrics-bromeliads-flies-final/weighted_unifrac_distance_matrix
